# Recent Advancements in Localization Technologies for Wireless Capsule Endoscopy: A Technical Review

**DOI:** 10.3390/s25010253

**Published:** 2025-01-04

**Authors:** Muhammad A. Ali, Neil Tom, Fahad N. Alsunaydih, Mehmet R. Yuce

**Affiliations:** 1 Department of Electrical and Computer Systems Engineering, Monash University, Melbourne, VIC 3800, Australia; muhammad.ali2@monash.edu (M.A.A.); neil.tom@monash.edu (N.T.); 2 Department of Electrical Engineering, College of Engineering, Qassim University, Buraydah 52571, Saudi Arabia; f.alsunaydih@qu.edu.sa

**Keywords:** wireless capsule endoscopy, WCE localization, RF-based localization, video-based localization, magnetic field-based localization, WCE tracking and safety

## Abstract

Conventional endoscopy is limited in its ability to examine the small bowel and perform long-term monitoring due to the risk of infection and tissue perforation. Wireless Capsule Endoscopy (WCE) is a painless and non-invasive method of examining the body’s internal organs using a small camera that is swallowed like a pill. The existing active locomotion technologies do not have a practical localization system to control the capsule’s movement within the body. A robust localization system is essential for safely guiding the WCE device through the complex gastrointestinal (GI) tract. Moreover, having access to the capsule’s trajectory data is highly desirable for drug delivery and surgery, as well as for creating accurate user profiles for diagnosis and future reference. Therefore, a robust, real-time, and practical localization system is imperative to advance the field of WCE and make it desirable for clinical trials. In this work, we have identified salient features of different localization techniques and categorized studies in comprehensive tables. This study is self-contained as it offers a comprehensive overview of emerging localization techniques based on magnetic field, radio frequency (RF), video, and hybrid methods. A summary at the end of each method is provided to point out the potential gaps and give directions for future research. The main point of this work is to present an in-depth review of the most recent localization techniques published in the past five years. This will assist researchers in comprehending current techniques and pinpointing potential areas for further investigation. This review can be a significant reference and guide for future research on WCE localization.

## 1. Introduction

Endoscopy is a medical procedure that involves the minimally invasive visual inspection of internal organs. The endoscope market is expanding as a result of the increasing elderly population and the prevalence of chronic diseases. Nonetheless, traditional endoscopy has certain limitations. The endoscope has a steerable tip that is manipulated externally via cable actuation. This mechanism spans the endoscope’s whole length, which may have a diameter of up to 13 mm, making it rigid. As a result, patients may endure pain, trauma, and discomfort throughout the treatment [1]. Patients are often advised to undergo sedation for endoscopic procedures, which makes many patients uncomfortable and leads them to avoid the procedure altogether. Moreover, given their semi-rigid characteristics, traditional endoscopes cannot access some regions of the GI system, including the small intestine. They are also inappropriate for long-term investigation because of the potential for infection and tissue perforation [2]. Furthermore, the COVID-19 pandemic has influenced the need for rigid endoscopes, as several scheduled surgical operations were postponed to focus on patients hit by COVID-19 [3].

WCE has emerged as an alternative to conventional endoscopy for diagnosing the GI tract. WCE is an innovative device that monitors the body’s interior organs through a capsule-sized camera. A standard WCE device has three primary components: a camera system for picture or video capture, a transmission system, and a battery that powers the internal electronics of the capsule [4,5]. WCE mitigates potential risks linked to traditional endoscopy by offering a sedation-free treatment that does not induce pain [6,7]. In contrast to traditional endoscopy, WCE is the gold standard for examining the small intestine [8,9]. Grand View Research estimates that the worldwide capsule endoscopy market was valued at around 483.8 million in 2022 and is anticipated to expand at a compound annual growth rate of 9.6% from 2023 to 2030 [10]. The increasing demand for capsule endoscopy is linked to the expanding worldwide population of elderly individuals, which reached 703 million in 2019 and is projected to climb by 1.5 billion by 2050 [11].

The existing locomotion technologies now in use do not have a practical localization system to manage the capsule’s movement within the GI tract safely. For safe navigation, an autonomous WCE system needs the real-time monitoring of the capsule’s trajectory and a control system allowing for continued navigation as a function of acquired data. Moreover, having access to the trajectory data of the capsule is highly desirable for creating accurate user profiles for diagnosis and future reference. To overcome these limitations, it is crucial to develop a precise localization system [12]. Furthermore, the effectiveness of follow-up interventions, remote surgeon access, and other medical procedures significantly relies on accurately and immediately determining the capsule’s location and orientation.

**Figure 1:**
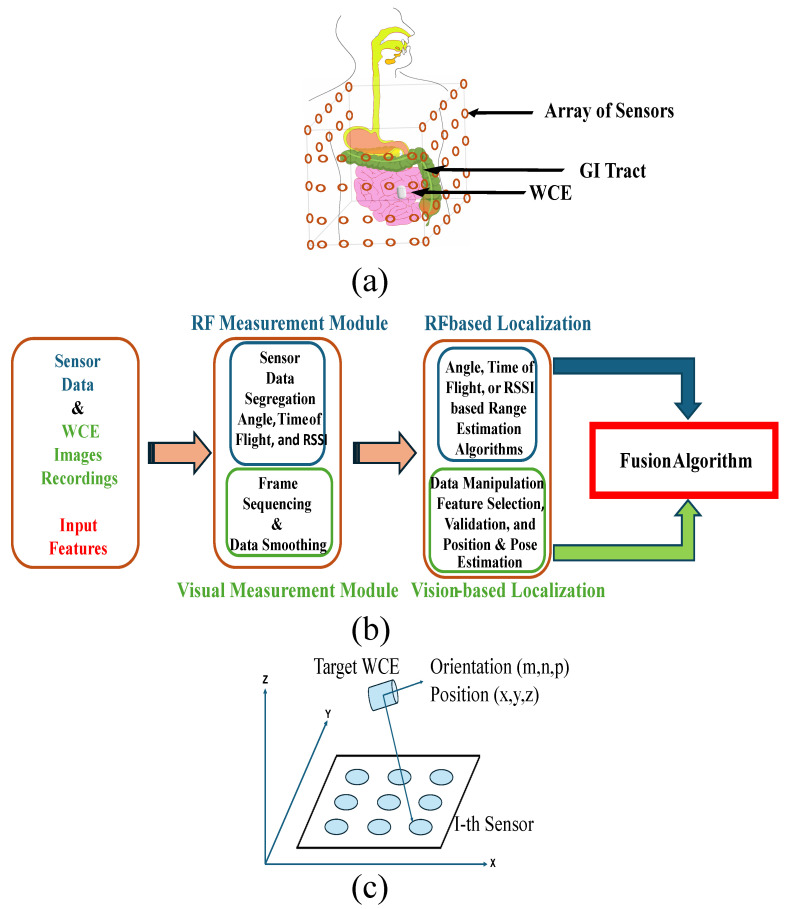
An RF and video-based hybrid localization technique. (**a**) A WCE device passing through a GI tract surrounded by an array of sensors. (**b**) Fusion of different measurement modules. (**c**) Position estimation.

However, in-body localization is challenging due to the body’s varied tissue composition, limited space within the capsule for additional circuitry, and interference from magnetic fields. Several techniques have been proposed to address these challenges, including radio frequency, magnetic field, and video processing-based methods. Figure 1 provides an overview of a hybrid localization technique that combines different methods. To create a practical localization system, researchers must address the following problems:

- The absolute position and orientation errors must not exceed 5 mm and 5°, respectively.
- The computational algorithms should be designed to be highly efficient with minimal processing complexity to achieve optimal real-time localization.
- Given its large-meter span, the GI Track’s relative distance error must be less than 5% to detect trajectory irregularities.
- The system should demonstrate resilience in everyday scenarios where ferromagnetic objects are present.
- The overall power consumption of the capsule’s and on-body/off-body excitation and data transmission circuitry should be low to support long monitoring.
- The system should be simple, lightweight, and easy to manufacture. To enhance integrability, it should use a minimal number of sensors.

**Figure 2:**
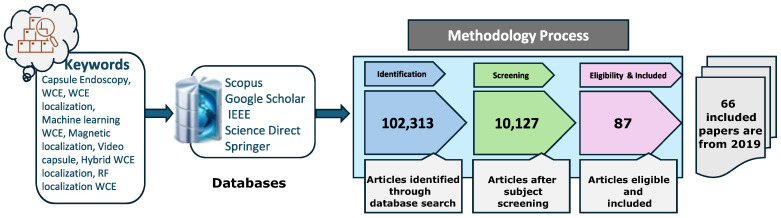
Review methodology.

**Figure 3:**
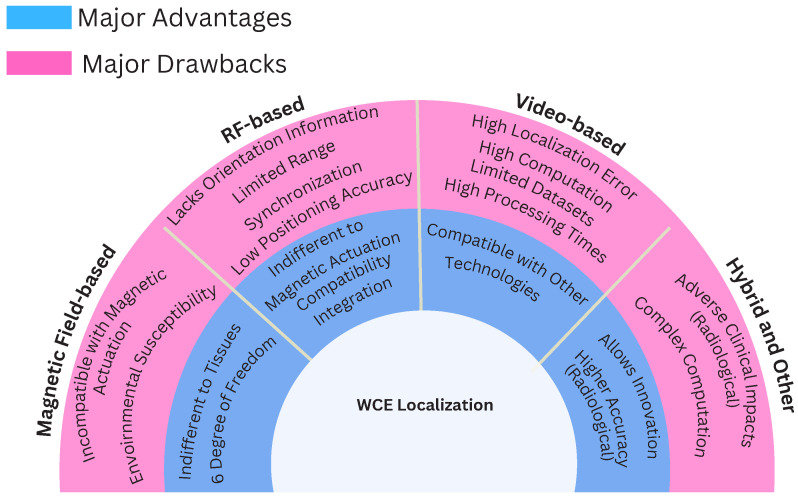
An overview of different techniques used for WCE localization.

The previous literature includes several notable studies on localization techniques [13,14,15]. However, in this study, the articles that were examined in detail were published after 2019, with a significant portion published in the previous few years. Figure 2 describes the rigorous research methodology adopted for the relevant latest papers selected for this review. We have identified salient features of different methods and categorized the studies in tables. Figure 3 presents an overview of the different categories of localization technologies. The review includes brief introductions of the latest methodologies used in the recent articles, the study environment, the type of hardware used, the reported localization error, and the way forward. Furthermore, this research presents a self-contained and comprehensive overview of localization strategies aimed at helping researchers understand current obstacles, possible remedies, and opportunities for improving the localization efficiency of wireless capsule endoscopy. In particular, the contributions of this work include a comprehensive updated review of magnetic, video, RF, and hybrid technologies-based localization techniques. This review can be a significant reference and guide for future research on WCE localization.

This paper is divided into sections: Section 2 reviews the latest advancements in magnetic field-based localization systems. Section 3 discusses emerging trends in RF-based localization techniques. Section 4 and Section 5 examine the most recent progress in video-based and hybrid localization techniques. Finally, in Section 6, we provide a conclusion. This study presents an in-depth review of the most recent localization techniques.

## 2. Magnetic Field-Based Localization Techniques

### 2.1. Introduction to Magnetic Field-Based Localization of WCE

Human tissues show varied reactions when exposed to electric and magnetic fields. While electric fields can lead to significant tissue damage and potentially death, even very strong static magnetic fields produce only minimal and difficult-to-detect effects on stationary tissues [16]. Magnetic field-based localization methods utilize the correlation between magnetic field strength and the proximity of a magnetic field sensor to the magnetic source. The most significant advantage of using magnetic fields for in-body WCE localization is their low interference in the complex heterogeneous tissue environment of the human body [17,18]. Moreover, unlike some RF-based methods, these methods do not depend on the line of sight between the target and the receiver/sensor and the composition of fat-to-muscle ratios of different bodies [19].

The magnetic flux density **B**(*r*) at a certain observer point *r* caused by the source magnet *s* can be approximated using the standard magnet dipole model [20]. When the Euclidean distance ‖*R*‖_2_=‖*r*−*s*‖_2_ is higher than the size of the magnetic source, the **B**(*r*) can be calculated as follows:

(1) $$B\left(r\right)=\frac{\mu_{0}\mu_{r}m}{4\pi }(\frac{3\langle O_{0},R\rangle R}{{\left\|R\right\|}_{2}^{5}}-\frac{O_{0}}{{\left\|R\right\|}_{2}^{3}})$$

where $\mu_{0}$ is the magnetic permeability in the vacuum, $\mu_{r}$ is the relative permeability of the surrounding tissue, which can be considered as 1, *m* is the source’s magnetic moment, and $O_{0}$ is the orientation of the source. Localizing a magnetic dipole involves solving for three positional and three orientation unknowns. If *N* sensors are outside the body, each sensor’s analytical B must be derived. Finally, the resultant non-linear equation system has to be solved for all six unknowns (position and orientation) by minimizing the error function *e* by using any non-linear estimation algorithm.

However, accurately localizing the WCE device using magnetic fields presents challenges, primarily due to environmental susceptibility. The limited volume of the capsule also restricts the use of stronger magnets or additional sensing circuitry. Moreover, the latest trends in developing practical WCE systems indicate the necessity of active locomotion systems. In magnetically actuated active locomotion systems, a small permanent magnet is embedded in the capsule, and the capsule’s motion is controlled by varying the external magnetic field using either a permanent magnet or an electromagnet [20,21,22,23]. Consequently, these external magnetic fields, along with the magnetic field of the components inside the WCE, can adversely affect the sensors’ readings. Magnetic field sensors must be calibrated before the start of the experiments to cancel out the Earth’s magnetic field and mitigate the effects of other magnetic, ferromagnetic, and electromagnetic (EM) materials in the experimental environment [24].

To minimize the impact of the interference, additional hardware in the form of multiple magnetic sensors and some embedded sensors, like IMU, is often desirable [25]. Various coil-based techniques have been developed to reduce the effect of magnetic field interference caused by active locomotion systems. Furthermore, the execution time must be minimized to facilitate real-time localization. In recent years, there has been a growing focus on utilizing multiple sensors, wearable localization systems, machine learning, and the combination of various algorithms to reduce localization errors. A magnetic tracking system typically consists of one or more magnetic sources, known as transmitters, and one or more sensor modules, known as receivers. Based on the static or time-varying nature of the magnetic flux, magnetic field-based localization can be divided into three main sections.

### 2.2. Static Magnetic Field-Based Localization

Static magnetic field-based localization techniques involve sensing static magnetic fields generated by a permanent magnet (PM). The magnetic flux detected by the sensors and the magnetic capsule’s localization coordinates can be determined using the dipole model [20]. The static magnetic field can be sensed by an array of sensors placed outside the body or by a Hall effect sensor placed inside the body. In recent years, significant advancements have been made in developing miniature sensors. Currently, miniature Inertial Measurement Units (IMUs) and 3-axis magnetic field sensors are extensively used. Researchers have frequently relied on neodymium magnets as PMs inside WCE due to their high flux density [26]. The main advantage of using a PM inside the WCE for localization is that the same WCE can be used for active locomotion by simply varying the external magnet field. However, these methods are affected by the geomagnetic flux density, which is of the same order as the PM’s flux density.

In [27], an analytical model for the 6 degrees of freedom (DoFs) magnetic localization of the WCE, equipped with an IMU and 3-axis magnetic field sensors and an external permanent magnet, is proposed. The WCE weighs 2.37 g with an overall dimension of 28.44 × 11.68 mm. The model uses a Jacobian-based iterative method to optimize the magnetic moment in the magnetic dipole model. Different translational and rotational motion modes were tested for localization in the experimental setup. The proposed analytical method shows great potential for real-time localization, with an average position error of 6.29 mm and an average orientation error of 2.93°. However, information on the power consumption and the transmission frequency of the WCE is required to assess its performance for long-term monitoring. Moreover, sensor drift and the limited accuracy of the magnetic field model could affect performance in prolonged operations or complex scenarios. Incorporating dynamic magnetic fields has the potential to enhance localization accuracy and minimize errors. Similarly, a novel 6 DoF localization system based on multi-sensor fusion for magnetically actuated WCE is proposed in [28]. To determine its 3D position and orientation, eight pose detection algorithms and two data fusion filters were analyzed. The capsule was embedded with an IMU, while the external system used a 5 × 5 Hall sensor array to compute the capsule’s position and orientation. A Bluetooth module was utilized to transmit IMU and video data. The direct and second derivatives methods were used to detect the pose and estimate the distance. Experiments were conducted within a work range (distance between the capsule and the Hall sensors) from 25 to 72 mm. A mean absolute error (MAE) was evaluated as 1.463 mm for the 3D position and 0.419° for 3D orientation. The capsule has overall dimensions of 29.5 mm × 13 mm, but information on its power consumption and weight is not provided. The magnetic shell used has a magnetic moment of only 0.65 A.m^2^, which generates relatively weak magnetic fields, resulting in reduced sensitivity. Miniaturizing the capsule and placing a magnet with a high magnetic moment can be beneficial for enhanced usability.

The fusion of different algorithms has been suggested in [29,30] to reduce the localization error. While algorithm fusion reduces localization errors, processing speed must meet real-time requirements. In [31], a hybrid model combines the magnetic dipole model and an analytical model for localization. Particle Swarm Optimization (PSO) utilizes a magnetic dipole model to provide an initial estimation, whereas the Levenberg–Marquardt (L-M) algorithm refines the estimates at high accuracy. The localization system reported an update rate of 30 Hz. The average position and orientation errors were reported to be 1.34 mm and 2.25°, respectively. However, the proposed method accounts for static and controlled dynamic movements but is less explored for unpredictable everyday scenarios. Furthermore, a closed-loop control mechanism can be developed to ensure precise navigation in the GI tract.

Magnetic localization by wearable sensors has recently drawn attention owing to their user-friendly nature. In [32], a variance-based algorithm was proposed as a wearable static magnetic localization to provide an optimal initial guess that reduced the number of iterations needed for sufficient localization, thus making the algorithm suitable for real-time applications with an update rate of 7.5 Hz. The experimental results for 16 sensors distributed across four faces of a cuboid reported an average positioning error of 9.73 mm and an average orientation error of 12°. The experiments were conducted in a controlled environment, assuming that the body would remain static. Further experimentation needs to be carried out in more realistic clinical setups to further improve the accuracy of the algorithm. In [33], a novel wearable permanent magnetic tracking system is proposed. It uses geomagnetic compensation and includes a 9-axis IMU, whose measurements are fused to calculate quaternion rotations by applying a fusion algorithm on its gyroscope, accelerometer, and magnetometer outputs. The system, validated with 36 magnetic sensors, achieved a 15-Hz update rate, an average position error below 2 mm, and an average orientation error of 5°. However, the 9-IMU must be positioned a minimum of 30 cm from the tracking area to prevent interference, hence increasing the device’s dimensions. Additionally, the current method requires periodic calibration to compensate for geomagnetic interference. A compact industrial-grade 9-axis IMU and investigating miniaturization methods can lead to a compact integration.

Magnetic field localization methods are susceptible to geomagnetic fields. Different techniques and algorithms have been developed to address geomagnetic interference. In [34], a novel differential signal-based approach was proposed to eliminate geomagnetic field interference without additional compensation methods. The approach utilizes a sensor array of 16 tri-axis magnetic sensors and a positioning-based optimization algorithm. It assumes that the geomagnetic field measured by closely located sensors is the same and calculates differential magnetic intensity between adjacent sensors. The method was validated through static and dynamic experiments. The reported results demonstrated that the method efficiently tracks the position and orientation of the WCE, with a mean position error of 5.2 mm in static experiments and approximately 7.5 mm in dynamic experiments. The system lacks wireless connection capabilities for transferring sensor data, potentially impeding its integration into completely portable or autonomous configurations. Additionally, the approach presumes that geomagnetic field variations between adjacent sensors are minimal. In real-world settings with considerable geomagnetic disturbances, this presumption may be invalid, potentially affecting accuracy. Improving the algorithm’s ability to handle significant geomagnetic disturbances and incorporating wireless data transfer would improve the system’s usability for dynamic, real-world scenarios.

Similarly, in [35], a differential static magnetic tracking method was proposed for geomagnetic interference compensation. This method pairs sensors in the array facing the same direction and cancels out homogeneous geomagnetic flux density by comparing measured values to analytical values. The authors concluded that 5 mm-long magnets balanced accuracy and capsule volume, with a mean relative distance and orientation errors below 4.3 ± 3.3% and 2 ± 0.6°, respectively. In [36], a multipoint simultaneous tracking method was proposed, which fuses multiple measurements to offset background noise. This algorithm reduces environmental noise impact by utilizing differences between measurements at different positions and optimizes the position and orientation by minimizing the error function between theoretical and estimated values. The approach, which completes calculations in 80 ms and compensates for patient movement, reported an average position error of 4.06 ± 0.29 mm and an average orientation error of 5.63 ± 4.24° for 5 DoF localization. However, the system can suffer when the rate of change in the background noise becomes higher than the sampling frequency. Furthermore, the impact of the nearby medical devices in a clinical setup needs to be investigated. Improving sensing capabilities and applying advanced filtering techniques can minimize the effects of transient noise, making the system more suitable for wearable applications.

### 2.3. Dynamic Magnetic Field-Based Localization

**Figure 4:**
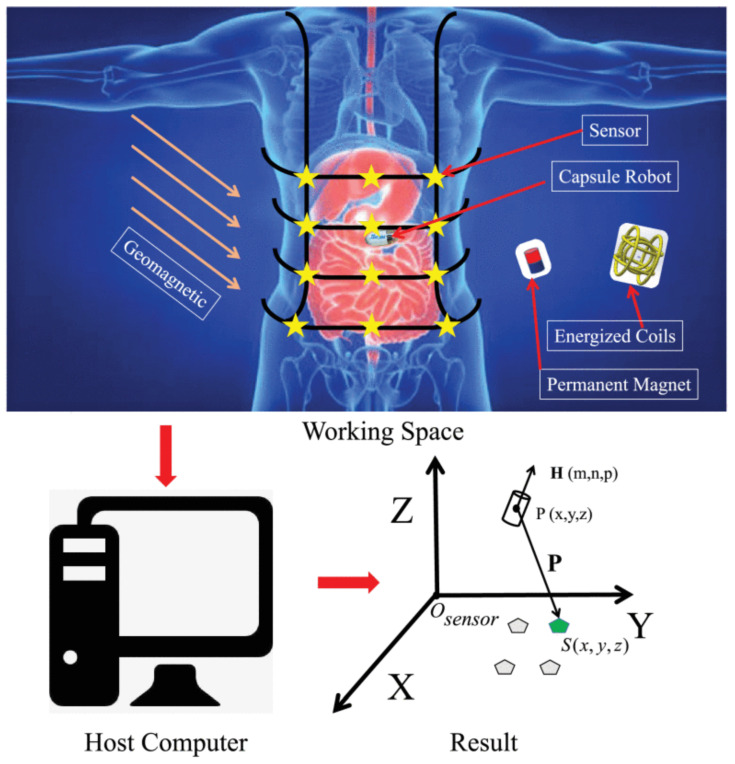
System overview of a magnetic field-based localization technique with active coils [36].

There is a growing trend in using EM coils supplied with an alternating current to generate a dynamic magnetic field, which a coil inside a WCE can sense. Figure 4 presents an overview of this localization method with active coils. Dynamic or quasi-static magnetic localization techniques can effectively eliminate geomagnetic interference by utilizing the time-varying magnetic fields generated by EM coils [37,38,39]. In [40], a compensation method was proposed to address relative movement using 12 tri-axial sensors to localize a permanent magnet inside the body. The method alternately switched two orthogonal reference coils on and off at a low speed. A new reference coordinate system was defined based on the orientations of these reference coils. The reported results demonstrate that the method achieved a mean position error of 3.8 mm ± 1.1 mm and a mean orientation error (MOE) below 3°. However, in the proposed system, the relative movement between the abdomen and the GI tract is not accounted for. Furthermore, the reference coils were rigid and localized successively. To enhance the system for clinical use, exploring the miniaturization of the reference coils for better integration and fixation on the abdomen could be beneficial. Additionally, optimized digital filters can be used for simultaneous coil localization.

Moreover, in [41], a hybrid 6-DoF active magnetic localization system for WCE with an extendable workspace is proposed. The system features an array of four electromagnets generating alternating magnetic fields at different frequencies, with the option to add more electromagnets to expand the workspace. The capsule includes a tri-axial Hall effect sensor for measuring magnetic field strength and a tri-axial accelerometer for measuring movement speed. For 3 DoF position estimation, sensor measurements are compared with analytical values from the model in [42]. For 3 DoF orientation estimation, pitch and roll angles are derived from the accelerometer, and the yaw angle is calculated from the difference between measured and expected magnetic field vectors. The system utilizes a two-stage optimization process: first, it uses ellipsoidal and spherical approximations for the magnetic field model; second, it minimizes the root mean square deviation between measured and estimated fields to minimize localization error. Despite the capsule containing several sensors, it manages to attain a position accuracy of 5 mm and an orientation accuracy of 5°. It can also be accommodated with an outer permanent magnet attached to a robotic arm for actuation. However, the external electromagnets operating at 70–100 Hz can consume up to 16 A current, suggesting a significant power demand, and the data are transferred from the capsule via a soft tether. Using neural networks to replace existing mathematical models can improve the accuracy of magnetic field approximations and enhance overall localization precision.

A scalable system adaptable to wearable or fixed configurations (e.g., jackets, toilet seats) has been proposed in [43]. The system comprises ingestible microdevices for the anatomical mapping of the GI tract (iMAG), comprises a Bluetooth Low Energy (BLE) microprocessor that handles communication at 2.4 GHz, a 3D magnetic sensor, and coin cell batteries with 2–4 weeks lifespan. The overall dimension of the iMAG is reported to be 20 mm × 8 mm. The proposed method employs magnetic field gradient-based localization using high-efficiency planar EM coils to generate 3D magnetic fields across a field-of-view of 40 × 40 × 20 cm^3^. The method reported a spatial resolution of 1.5 mm and a temporal resolution of 300 ms. The system was validated on large animal models and demonstrated high spatial resolution and safety for non-clinical applications. However, the system’s communication range is constrained to less than or equal to 50 cm when the capsule is deep inside the GI tract. Moreover, the high attenuation of 2.4 GHz Bluetooth signals by body tissues, especially in areas with thick gastric and intestinal walls, is a significant issue. Additionally, another drawback of such techniques is the power-intensive external magnetic coils that need to be activated and deactivated in sequence, leading to inefficiencies in power usage. For future studies, it may be beneficial to use lower frequency bands, such as around 400 MHz or 915 MHz. These lower frequencies experience less tissue absorption, which can result in stronger signal reception.

### 2.4. Magnetic Induction-Based Localization

Battery life is critical in any WCE system, as there is always a risk that the battery may be depleted before the examination of the gastrointestinal tract is complete. Consequently, wireless power transfer (WPT) methods, particularly Inductive Power Transfer (IPT), are gaining popularity in capsule endoscopy. Coil grid methods are particularly favored because they enable the localization of a receiver (RX) coil within the body while simultaneously transferring power inductively. In these methods, the distance between the transmitter (TX) grid and the receiving coils is estimated by calculating the mutual inductance between them using the Neumann Formula [44].

(2) $$M=\frac{\mu_{0}}{4\pi }\int_{C_{1}}\int_{C_{2}}\frac{{\mathrm{dl}}_{1}\cdot {\mathrm{dl}}_{2}}{r}$$

where *M* is the mutual inductance between the TX and the RX coils, $\mu_{0}$ is the permeability of the free space, $C_{1}$ and $C_{2}$ are the paths of the coil 1 and coil 2, $dl_{1}$ and $dl_{2}$ are the differential lengths along the electrical paths for the current $I_{1}$ and $I_{2}$ of the TX and RX coils, respectively, and *r* is the distance between $dl_{1}$ and $dl_{2}$. The dot product ${\mathrm{dl}}_{1}\cdot {\mathrm{dl}}_{2}$ represents the angle between the differential length elements. The distance can be estimated based on the reflected impedance on the TX grid caused by the implanted RX coil. The RX coil induces a higher reflection impedance in the coils of the grid that are closer to it. Consequently, the location of the coil with the highest reflection impedance is used to estimate the RX impedance.

In [45], a dual-purpose Wireless Power Transfer (WPT) system was introduced to power and localize a capsule within the GI tract. The system features an 8 mm RX coil inside the capsule and two TX coils, each consuming 5 W outside. The TX coils are orthogonal printed spiral coils designed for uniform magnetic field generation, while the RX coil is a miniaturized 3D cross-type coil that enhances power reception and localization. Using magnetic resonance between TX and RX coils, localization is achieved by measuring power variations at different positions within a simulated GI tract environment. The maximum localization error reported is 12%, equating to ±13 mm within a 100 mm × 100 mm × 100 mm volume. Although the simulation demonstrates a low specific absorption rate (SAR) value at a frequency of 5 MHz, with each coil supplied with 5 W, the total power consumption of 10 W renders the system energy inefficient, especially when used as a wearable device. For future work, reducing translational misalignment and wide coverage by increasing TX coils can ensure better alignment and tracking. Moreover, TX coils can be made more energy efficient to make the technique more suitable for wearable purposes.

In [46], a system utilizing quasi-static mutual inductance (QS-MI) for precise localization is proposed. The method uses a tri-polar plane-type (TPT) coil system on the primary side and orthogonal coils on the ingestible. The system is validated through simulation and experimentation. Mutual inductance is measured between the primary and secondary coils using a resonance-based circuit architecture and vector network analyzer (VNA). The simulation process focuses on evaluating the system’s performance under realistic conditions by introducing quantization and thermal noise. The noisy signals are processed using estimation algorithms like least squares estimation (LSE) to recover the MIs. Finally, localization is performed using an optimization algorithm based on these MIs. The experimental setup uses a Vector Network Analyzer to measure the transmission coefficient between TPT coils and secondary miniature coils, calculating mutual inductance in both air and saline environments. The localization is performed using the objective function and solving it using linear steepest descent (SD). An accuracy of less than 1 cm has been reported. The accuracy can further be improved by utilizing different configurations and sizes of the transmitter coils. Moreover, advanced tracking algorithms, such as particle filters, can be investigated for initialization.

In [47], an EM tracking system (EMTS) was proposed to determine the 5D pose of a tiny induction coil, including 3D position and 2D orientation. Unlike traditional systems with wired connections, this system uses wireless communication, enhancing flexibility for medical and industrial applications. It employs nine-channel sinusoidal signals for the simultaneous excitation of transmitting coils, improving system effectiveness. Sophisticated algorithms, including the L-M method and PSO, are used for optimization and initial positioning. The system reported an average location error of less than 2.3 mm and an orientation error of 0.2°. Accuracy may be affected by the overheating of the power amplifiers due to signal fluctuations, which can affect the tracking precision. High-frequency noises that interfere with the induction process can be filtered to achieve better signal clarity and tracking precision.

Recent advancements in on-chip sensing have shown that magnetic sensing can be accomplished with low power consumption and a compact design. The 3D position tracking system introduced in [48] has reported a tiny size sensor (1.06 mm^2^) fabricated using Complementary Metal Oxide-Semiconductor (CMOS) 65 nm technology with a power consumption of only 6 mW. The integrated circuit includes an on-chip inductive coil, an analog front end (AFE), a Delta-Sigma Modulator for data acquisition, an output data driver, and voltage regulator circuits, among others. The on-chip multi-layered coil detects the magnetic field generated by eight external transmitter coils at specific frequencies (20–34 KHz). The data acquisition and processing leverage the Levenberg–Marquardt non-linear least squares algorithm for position and pose estimation. The sensing mechanism was tested in benchtop and pre-clinical settings, demonstrating precise tracking in dynamic real-world contexts, including live porcine airways. The method reported a navigation accuracy of 1.1 mm for 5-DoF tracking. For 6 DoF, it achieved a position accuracy of 0.8 mm and an angular error of 1.1°. This performance was recorded within a volume of interest measuring 15 × 15 × 15 cm^3^. The results were gathered at an update rate of 20 Hz, and the pre-clinical tests in live swine airways demonstrate its practical application and precision with a reported worst-case registration accuracy of 5.8 mm. Furthermore, CMOS fabrication processes are robust and allow for mass production, making the sensors scalable for widespread and the reported sensor costs around USD 1.50, compared to traditional 5-DoF sensors that cost USD 25 and 6-DoF sensors that cost USD 250.

The on-chip sensing method presented in [49] uses two micro-chip devices that use Hall-effect sensors to measure the magnetic field present and digitize the information before transmission, which can minimize the conversion complexity. The testing device was fabricated in 65 nm CMOS technology and also includes a 3D magnetic sensor and an inductor coil with a footprint of 3 × 3 × 0.75 mm^3^. The system uses planar electromagnetic coils that create 3D magnetic field gradients in a 20 × 20 × 10 cm^3^ field of view that can be further scaled. The reported localization errors are less than 100 μm with an update rate of 7 Hz, and the chip is powered up wirelessly using WPT technology at 13.56 MHz. The same frequency is used for data telemetry. The reported current consumption is 2.2 and 1.5 mA for low-noise and low-power modes, respectively. Comparatively, in [50], a 3D magnetic sensor with on-chip orthogonal coils in 65nm CMOS generates an induced electromotive force (EMF) when exposed to the AC magnetic field. The reported sensor is 4 mm^2^ in and consumes negligible power, 14.8 μW, to achieve less than 1 mm 3D positional accuracy. However, further detailed experimentation in clinical settings is necessary to evaluate its efficiency and reliability for endoscopic applications.

A low power frequency division-based localization method with sub-mm precision has been reported in [51]. The reported frequency-division multiplexed magnetic localization (FDMML) technique uses frequency-division multiplexing to assign a different offset frequency to each external magnetic beacon. It enables them to operate simultaneously, eliminating sequential beacon activation. Augmenting the excitation frequency presents various notable benefits, such as a decrease in coil dimensions and an enhancement in the voltage acquired by the receiver coil on the sensor, resulting in a more excellent signal-to-noise ratio. An on-chip resonant 2.048 MHz receiver coil senses the voltage from six external magnetic beacons, and the system utilizes Welch’s method for frequency analysis and ANNs for spatial position reconstruction. The proposed chip has an area of 1.4 mm^2^ and consumes a maximum total power of 336 μW. The system reported less than 1 mm localization error, with localization circuitry alone consuming 247 μW. However, the system has not yet been tested in clinical trials, limiting its validation for practical use in GI diagnostics.

### 2.5. Summary of the Magnetic Field-Based Localization Techniques

Emerging technologies have shown promising results in accurately localizing WCE using static or dynamic magnetic fields, achieving position and orientation errors under 5 mm and 5°, respectively. Techniques often involve induction coils, IMU, and tri-axial sensors within the capsule. However, the limited volume of commercial capsules poses a significant constraint. Static magnetic field-based techniques can use IPM (internal permanent magnet) to occupy between 10% and 30% of the available space of the capsule. The methods discussed in [27,28,41] take up significant capsule space owing to the usage of additional components used for localization purposes compared to approaches that solely utilize IPM, as seen in [32,34,35]. Recent advancements in the use of 65 nm CMOS technology demonstrated that compact sizes are achievable. For real-time localization, minimizing computation time is crucial. Advanced machine learning techniques and various algorithm combinations can help to reduce computational time for effective real-time monitoring. The update rates reported in [33,36] are 67 ms and 80 ms, respectively. This boost in calculation speed stems from the fusion of various algorithms.

There is still a pressing need for a real-time localization system compatible with an active locomotion system. Dynamic magnetic field-based localization techniques have gained immense popularity in recent years due to their effectiveness in canceling environmental magnetic field interference. These quasi-static magnetic field-based methods are ideal for localization systems compatible with active locomotion systems while achieving low localization errors. Onboard sensing and wireless transmission through IMU and magnetic sensors can negatively impact the limited battery resources. However, recent on-chip magnetic sensing solutions have demonstrated that carefully designed chips and energy-efficient methods can keep the WCE operational for several weeks [48].

Wireless Power Transfer (WPT) can be a promising solution for power transfer and estimating the capsule’s location. Combining precise localization with effective power control enhances GI tract inspections. Wireless Power Transfer (WPT) extends capsule operational duration by recharging or directly powering the capsule, overcoming the limitations of traditional internal batteries and enabling thorough, extended diagnostic procedures.

The use of additional sensors, either within the WCE or in the localization system, can improve localization accuracy but may make the system impractical. The localization methods in [28,32,33,35,40] reported excellent localization accuracies, but the use of a higher number of sensors can make the overall system unsuitable for practical use. Future research should focus on using a minimal number of sensors and localization equipment inside the capsule and in the surrounding environment while emphasizing efficient and real-time algorithms. Moreover, wearable localization systems are highly desirable as they can assist patients in daily activities during extended examinations lasting up to 12–14 h. These systems have achieved low localization errors, but further improvements are required for commercial use. To prevent damage or discomfort to the abdomen, an efficient wearable transmitter is desireable. The system must be power-efficient and robust enough for daily activities. Consequently, magnetic localization for capsule endoscopes is an ongoing area of research and is not yet suitable for commercial capsules. Table 1 summarizes the emerging magnetic field-based localization techniques along with WCE Components (WC) and external components (EC) for quick reference and comparison between different techniques.

## 3. RF-Based Localization Methods

### 3.1. Introduction to RF-Based Localization Techniques

Wireless systems rely on EM materials of different frequencies for communication. Each wireless transceiver uses a specific part of the EM spectrum to transmit and receive signals. EM-based localization has been a critical area of research since the advent of wireless technology. Modern mobile communication, made possible by cellular networks, relies on the phone’s location to maintain a stable connection over longer distances. Technological advancements in miniaturization and application-specific integrated circuits (ASICs) have enabled the development of circuits as small as nanometers, which can be implanted inside an animal’s body [52]. In-body communication occurs at specific frequencies that can penetrate the body’s complex muscle and fat environment. Medical implants communicate with external devices using different frequency bands. However, unlike off-body EM wave propagation, the in-body propagation of EM waves is complex. The characteristics of a traveling EM wave change with the relative permittivity of the medium it traverses. Since an animal’s body consists of different layers of varying materials, an EM wave must pass through layers with differing permittivities and electrical properties as it travels inside and outside the body [53].

Consequently, an RF-based localization system depends on the properties of the wireless propagation channel, such as attenuation, relative permittivity, and multipath characteristics. In a complex environment, an EM wave transmitted by a TX antenna undergoes reflection and diffraction before reaching the RX. Due to these processes, multiple copies of the transmitted signals are received at the RX, a phenomenon called multipath propagation. These signal copies arrive at the RX with varying amplitudes, arrival angles, times, and phases [52]. As a result, EM waves traveling from a TX to an RX have various characteristics: Angle of Arrival (AoA), Time of Arrival (ToA), Direction of Arrival (DoA), and Received Signal Strength Indicator (RSSI). Researchers have utilized these properties to determine the implant location within the body with various techniques. In the next section, we will describe different approaches and methodologies that have been developed related to RF-based localization systems and discuss the existing research gaps within this area of research.

### 3.2. Advancements in RF-Based Localization Techniques

Recently, several new techniques have been proposed for RSSI-based localization methods. When a signal is transmitted from within the body, it attenuates because of the structure of relative permittivity and electrical conductivity. Diverse techniques and path loss models use these attenuations to determine the transmitter’s or TX’s position. Algorithms that use RSSI information are distance-dependent or distance-independent. A distance-dependent algorithm involves triangulation, trilateration, LS, and MLE methods [54]. On the other hand, distance-independent algorithms find the location of the TX by selecting signals with particular strengths and computing the geometric centroid of the RXs. These methods approximate the capsule’s location using a path loss model on the RSSI values. The path loss in dB at a distance (d) from the in-body TX is modeled as follows [55]:

(3) $$PL\left(d\right)=10nlog_{10}\left(d/d_{0}\right)+PL\left(d_{0}\right)+X_{\sigma }$$

where $d_{0}$ is the reference distance, *n* is the environment-dependent path loss exponent that should increase in lossy environments, and *X* is the random scatter around the mean σ. It describes the deviations caused by varied propagation materials and antenna gain in different directions.

Current research advances concentrate on integrating statistical models, artificial intelligence (AI), circularly polarized antennas, antenna arrays at the reception side, and machine learning (ML) techniques to minimize localization errors. A method for the precise localization of wireless capsules using machine learning and smoothing path loss with capsule exact positioning in the small intestine was proposed in [56]. The authors utilized two path-smoothing techniques: local linear regression moving average (LLRMA) and locally weighted linear regression (LWLR). They treated both the smoothed data and the scattered data as input features of five regression algorithms using machine learning: Decision Tree (DT), Random Forest (RF), Extreme Gradient Boosting (XGB), Linear Regression (LR), and K-Nearest Neighbors (KNN). Reportedly, this methodology optimizes the input features by manipulating data, selecting features, and using k-fold cross-validation to ensure accuracy. The methods reported a localization accuracy of less than 0.22 mm using 48 sensor RXs. The simulations were conducted in the ultra-wideband (UWB), 3.1–6 GHz, and medical implant communication service (MICS), 402–405 MHz, frequency bands. Hardware implementation and practical deployment scenarios had to be explored to gauge the method’s feasibility in clinical trials.

In [57], an interesting method for correcting position estimation errors in capsule endoscopy is introduced. The approach uses the trilateration method and includes two error correction techniques. Firstly, it adjusts RSSI values based on their magnitude pattern for a temporary estimate. Secondly, it corrects RSSI reductions caused by the angle between the capsule and the receiving antenna. The proposed algorithm reported an estimation error below 40 mm for 92% of simulation points and 89.7% of experimental points at 433.92 MHz. The proposed algorithm lacks accuracy where the received signal strength measurement does not align with the antennas. Additionally, in some cases, intersections in the trilateration method are not possible and require additional corrections. In [58], localization is proposed at 17 MHz using EM scattering based on a sparse vector reconstruction (SVR) algorithm. The simulation evaluates the method’s performance by calculating the mean localization errors and comparing the results obtained from the SVR technique with the conventional Moore–Penrose inverse solution. The system predicts that using more than 10 RXs can achieve a localization error of less than 2 mm, but only at frequencies below 17 MHz. Experimental validation is required to assess the validity of the system in real and complex environments.

The human body’s intricate and diverse EM properties can influence signal propagation. The diagnostic technique can be tailored to a patient’s unique anatomical and physiological variations through surface field modeling. In [59], the localization approach is based on analyzing RF signals, specifically using a Hertzian dipole radiator operating at 433 MHz inside the human body. It aims to estimate the intensity of the surface field by considering the location and direction of the dipole. An analytical solution for calculating the surface field intensity is derived based on the capsule’s orientation. In [60], the work focuses on localizing wireless capsule endoscopes using a hybrid approach incorporating one-shot learning and trilateration methods with an operating frequency range of 3.75–4.35 GHz. Trilateration is employed for accurate distance measurements, while one-shot learning, facilitated by a Siamese neural network (SNN), adapts to the varying tissue features found in different areas of the GI tract. The collected channel data are based on the Laura Human Voxel Model to mimic the in-body environment. A zone-specific path loss model that considers the varying anatomy of the GI tract is utilized. Different zones are created using channel frequency response (CFR) measurements, and the SNN method is used to determine the specific zone based on real-time CFR values. After identifying the zone, a non-linear least-squares-based trilateration method is used for localization, resulting in a mean distance error of 26.44 mm when the system is configured with three zones. However, conducting real-world validations is critical for confirming the methodology’s robustness and reliability.

**Figure 5:**
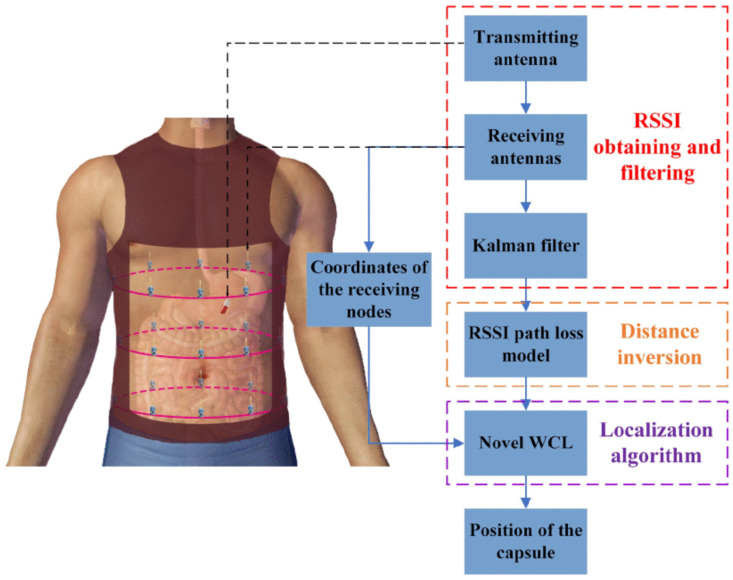
System overview of a wearable capsule endoscope electromagnetic localization system [61].

Centroid localization algorithms are distance-independent, and their low computational complexity and low power requirements make them ideal for WCE localization. In [61], a novel weighted centroid localization (WCL) algorithm was proposed, where a scaling factor is used to assign weights (see Figure 5). The authors validated the algorithm by implementing 24-array receiving antennas and reported a localization root mean square error (RMSE) of 36.3 mm, with a minimum localization error of 21.9 mm, 17% lower than conventional WCE algorithms. Similarly, in [62], a Smoothed Path Loss Degree (SPLD)-based WCL algorithm was proposed to enhance the accuracy of capsule endoscopy using a 3D sensor array of miniaturized impulse radio (IR) UWB transceivers to receive RSSI values. The novelty of the SPLD-WCL algorithm lies in its ability to effectively manage path loss variations and improve localization accuracy using a weighted centroid method based on the degree of path loss. The results reported that the SPLD-WCL method achieved high precision with an RMSE of 6.83 mm. However, the results are based solely on simulations, and experimental validation is necessary to confirm the performance of the proposed method.

The Phase Difference of Arrival (PDoA) models are being researched intensively because the phase of signals from implants remains relatively stable compared to the RSSI value in the presence of different tissues [63]. This method does not require knowledge of the electrical properties and distribution of individual tissues inside the body and can be accurately applied to patients with varying tissue thicknesses [64]. Based on the phase difference at the receiving antennas, the distance of the capsule *d* from an antenna on the human body can be calculated as follows:

(4) $$d_{i}=\frac{c}{2\pi \sqrt{\epsilon_{r}}}\times \frac{\delta \phi_{i}}{\delta f}$$

where $d_{i}$ is the distance between the capsule and the *i*-th antenna on the body, *c* is the speed of light in vacuum (299,792,458 ms^−1^), $\epsilon_{r}$ is the relative permittivity, $\delta \phi_{i}$ phase difference between the two receiving frequencies, and δf is the difference between the two frequencies. Once the distance is determined, any linear or non-linear least squares method can be applied to obtain the capsule coordinates.

In [63], an adaptive phase detection algorithm is proposed to improve localization accuracy using a helical antenna and an adaptive body permittivity model. The proposed capsule utilizes the phase information analysis of a signal transmitted through a helical antenna for position determination. Reportedly, the permittivity of the adaptive body model dynamically adjusts itself to compensate for the in-homogeneity and heterogeneity in human tissues and propagation based on the different locations of the capsule. It involves the iterative refinement of the PDoA and Gauss–Newton algorithms for localizing. Validation has been carried out with computer simulations and phantom experiments to simulate the electrical tissue characteristics of human tissue. The authors reported that combining the algorithm and the helical antenna improves the accuracy by around 30% compared with a homogeneous body model. The average error of 16 mm with five RXs has been reported.

In [65], a hybrid phase detection method with a dynamic model adaptation strategy has been proposed to find the location of the WCE. The algorithm uses the phase differences of the signals acquired at different frequencies to give the actual possible placement of the capsule. Afterward, an iteration refinement technique using Gauss–Newton optimization is adopted to update model permittivity values for accuracy enhancement. The procedure reported a 15% localization error decrease with a value of 12 mm compared to the traditional methods that rely on models with dielectric constant properties.

Of the least complicated methods to obtain the range of an object, DoA/ToA is one of them. The idea is to measure the time a signal travels from the target to several RXs with known coordinates [66,67]. DoA-based estimation is based on the principle that only a signal arriving from the estimated direction is focused upon, and signals coming from other directions are neglected. Given that the distance d is between the target and an RX, the distance can be computed using the following equation:

(5) d=ToF×v

where ToF is the time of flight of the signal from a target TX to a known RX, and *v* is the speed of signal propagation:

(6) $$v=\frac{c}{\sqrt{\epsilon }}$$

where c is the speed of light in a vacuum and ϵ is the propagation coefficient. Given the spatial location distribution of the RXs and their distances from the TX, different trilateration or triangulation methods may be utilized to estimate the location coordinates of the TX. This is essentially a method that has mainly been applied in homogeneous environments, like free space, where the propagation speed of the signal remains invariant due to the prevalence of a single medium [68].

However, in the case of WCE, the capsule travels through the human body, which consists of tissues with varying permittivities. These differences in permittivity affect the signal propagation speed, leading to errors in calculating *d*. To address this issue, a ranging error model for WCE localization was proposed in [69]. The average permittivity of the human body model [70] is used to calculate the propagation speed. A 3D simulation model of the human torso was designed, and using a set of 32 RXs, the average localization error for ToA and RSSI methods was evaluated. The RSSI-based method resulted in a mean localization error of more than 48 mm, while the ToA-based technique reported a mean localization error of 15 mm. Although DoA/ToA-based methods provide low localization errors, they are not feasible for near-field applications due to the high speed of EM waves, i.e., approximately 3 × 10^8^. One of the most challenging aspects of one-way ToA-based localization in WCE is achieving precise time synchronization between the capsule’s TX and the external RXs.

Poor synchronization can lead to inaccurate Time of Flight (ToF) calculations, resulting in distance estimation errors and adversely affecting localization accuracy [71]. Moreover, due to the multipath effect caused by the heterogeneous nature of the human body, the received signals may become distorted, complicating the DoA/ToF models. Two-way ToA measurement systems can be employed to mitigate synchronization issues. These systems generally consist of an array of RXs mounted in a 2D plane or circular configuration around the body, wherein DoA estimation is achieved by using various signal processing algorithms. A hybrid approach incorporating the Extended Kalman Filter (EKF) method with DoA/ToA and IMU data for further enhancement in localization accuracy is proposed in [72]. In the paper, the authors used a circular array of antennas with different numbers of elements and developed and assessed the RMSE. The authors concluded that localization performance significantly improves if the number of antenna elements is kept below 16.

Radio Frequency Identification (RFID) technology involves placing RFID tags inside a wireless capsule and communicating with RFID readers. These tags can be either active or passive. Passive tags are more compact, lighter, and less expensive than active ones; however, their utility is limited due to a limited communication range, storage capacity, and computing power. In contrast, active RFID tags have a battery source, allowing them to transmit data at fixed intervals. The signal power and transmission time information are used to determine the location of the tags within the body. In [73], an array of antennas is arranged in 3D around a body containing an RFID tag, with antennas placed 3 cm apart. An algorithm estimates the tag’s location using gravity, relying solely on the proximity of the antennas that can detect the tag rather than on RSSI. The reported mean error in location estimation is 20 mm. In [74], a similar antenna array is proposed, but localization is based on the assumption that the RF signals transmitted by the RFID tag are symmetrical. The algorithms utilize external RXs positioned symmetrically to detect these signals, forming areas on the symmetric faces of the RX arrays. By considering these areas’ similarity in position, shape, and size, accurate 3D positions of the signal source can be determined through back projection calculations. The reported position error is 5 mm in the x and y directions, with a total position error of 20 mm.

### 3.3. Summary of RF-Based Localization Techniques

The RF-based localization system leverages the properties of the wireless propagation channel, including attenuation, relative permittivity, time, and multipath characteristics. EM waves experience reflection and diffraction in complex environments, leading to multipath propagation. As a result, different copies of the transmitted signals exhibit varying amplitudes, angles of arrival, times of arrival, and phases, creating EM waves with distinct characteristics. Recently, there has been a growing emphasis on exploring various combinations of optimization algorithms, AI, circularly polarized antennas, arrays of antennas on the receiving side, and ML techniques to minimize localization errors. Due to limited battery capacities, coil grid methods are becoming increasingly popular, as they facilitate the localization of an RX coil within the body while inductively transferring power. However, technical challenges, such as the need for line-of-sight, complicate the implementation of these technologies.

Additionally, during practical trials, antennas are typically attached to the patient’s body, which serves as a reference point for localization. It is important to note that this reference body is also in motion. The body’s movement and the involuntary motion of internal organs contribute to higher localization errors. Implementing ToF, ToA, DoA, and AoA methods in real-time settings is much more challenging due to the high speed of EM waves. Furthermore, RF-based methods cannot determine the orientation of the capsule.

Emerging trends in RF-based methods include integrating diversity in AI, ML, and optimization algorithms. This area can be further explored by combining different combinations of RF-based technologies with video and magnetic field-based localization methods. RF-based techniques can localize the capsule without any additional hardware, and along with video or magnetic field-based techniques, the capsule’s orientation could also be determined. Recent research has also shown that a fusion of multi-DoF IMU sensor data with RSSI data can lead to more accurate results. Table 2 offers an overview of the differences among various RF-based techniques and highlights recent advancements in the RF-based localization field.

## 4. Video-Based Localization Techniques

### 4.1. Introduction

Video-based localization approaches have followed a growth trend over the years, part of which is influenced by AI and ML. The means through which AI can be integrated into WCE enable the development of intelligent algorithms that enhance localization accuracy and predict disease patterns, thus changing patient care scenarios. This integration poses challenges in sensitivity and intuition but presents significant steps to achieving video-based localization with increased precision. This kind of advancement enables precise spatial information regarding detected abnormalities in the GI tract. Deep learning-type Convolutional Neural Networks (CNNs) can efficiently analyze visual data, and the principal component analysis (PCA) reduces the dimensionality for endoscopic images to extract the essential features, which results in the enhanced accuracy of localization algorithms [75,76,77,78]. The core operation of CNNs is convolution, expressed as follows:

(7) $$f\left(x,y\right)=\sum_{i=-a}^{a}\sum_{j=-b}^{b}g\left(i,j\right).h\left(x-i,y-j\right)$$

where f(x,y) is the output image, g(i,j) is the kernel, and h(x−i,y−j) is the input image. This operation allows CNNs to extract high-level features from images for accurate pattern recognition and localization [77,78].

Video-based localization faces significant challenges in obtaining high-quality images from WCE [75]. The dark areas in frames and blurry photos due to poor illumination conditions and the lack of a controlled locomotion system make it challenging to capture vital information, leading to lower accuracy in vision-based localization systems. Furthermore, the constant movement of the capsule inside the intestine makes it even harder to localize its orientation and pose. Many pre-processing processes have been developed to overcome these challenges and make the captured videos more useful for localization. During the last few years, machine learning-based algorithms have been developed to enhance the captured frames’ features and localize the WCE based on the captured video frames [76]. It is crucial to thoroughly analyze and compare existing techniques to address commercial capsules’ technological challenges. This will pave the way for future research in this domain and create opportunities for improvement.

### 4.2. Recent Developments in Video-Based Localization Techniques

In the past few years, different algorithms and methods have been proposed for localizing various lesions and detecting bleeding inside the GI tract. An Attention Aware CNN algorithm and ResNet-50 as a convolutional stem are utilized in [77], which used public Bleeding and Kvasir-Capsule datasets to localize bleeding with 95.1% and lesions with 94.7% accuracy. This highlights the effectiveness of attention mechanisms in medical imaging, although there remains a potential research gap in real-time processing capabilities. Similarly, a deep homography-based localization method that used a MobileNet-based CNN algorithm is reported in [78]. The model estimates frame transformations through 4-point homography parameterization, focusing on the displacement of four corners between frames. The displacement is subsequently converted from pixel units to millimeters using the predetermined average diameter of the small intestine. The reported Mean Absolute Error (MAE) of the computed displacements compared to the reference values obtained from the Rapid Reader software, *Given Imaging* proprietary software (Rapid V8), was 4.87% ± 4.12%.

Additionally, for polyp localization, a modified R-CNN method utilizing ResNet-50 and ResNet-101 models with data augmentation and fine-tuning is proposed in [79]. This system uses a region-based convolutional neural network modified on still frames to identify locations for polyps by generating masks around them to indicate the precise location. The polyp images are represented using pre-trained Resnet-50 and Resnet-101 models as feature extractors. In order to increase detection accuracy, the used models are fine-tuned through multiple publicly available polyp datasets; these include CVC-ClinicDB, CVC-ColonDB, CVC-PolypHD, and ETIS-Larib. The proposed methodology has demonstrated great potential in improving polyp detection and localization in a clinical setting. Using the ResNet-101 backbone and Balloon pre-trained weights, the best performance on the WCE dataset has been achieved with an F1 score of 96.6% and an F2 score of 96.10%. This showcases the effectiveness of the methodology and its ability to enhance clinical diagnoses. However, the computational cost is high due to the use of separate graphic processing units. The use of annotated data can further improve the accuracy of the method.

One of the efficient localization methods for temporal abnormalities in long WCE videos is the use of Graphical CNN. This helps to reduce the time physicians use for reviewing as it identifies anomalies precisely and accurately without requiring frame level annotations. WCENet is another deep CNN that achieves 98% accuracy in classifying and localizing anomalies in the gastrointestinal tract due to an attention-based mechanism that it incorporated with a customized SegNet [80]. The study utilized the KID dataset for WCE images. It used a hybrid anomaly localization method, combining Grad-CAM++ and SegNet, which allows for the precise identification and segmentation of abnormal regions, resulting in high accuracy and precision. However, its implementation may require substantial computational resources, potentially limiting its application in real-time or resource-constrained environments. Additionally, the performance of such a model depends so greatly on the quality and diversity of its training data, which may also influence generalizability to new or unseen cases that are not especially well-represented in the KID dataset.

Algorithms for dimensionality shift were analyzed in order to provide better post-processing image analysis for images obtained from WCE examinations. Specifically, Oriented FAST and Rotated BRIEF–Simultaneous Localization and Mapping have realized data processing for 2D WCE images in a video sequence and utilized the Shape-from-Shading algorithm to reconstruct a 3D model [81]. The algorithm reconstructs the environment and localizes the camera by comparing pairs of points between images. For the ORB-SLAM, the study constructed the 3D representation of the bowel wall and localized the WCE with a mean absolute error ranging from 4.1 to 3.9 cm. This showed that the algorithm could realize accurate localization and detailed 3D modeling for WCE examinations.

Furthermore, with the advancement in micro-electromechanical systems (MEMS), several techniques have been proposed for wireless capsule endoscopy, involving multiple components integrated inside the capsule. Specific feature point tracking techniques have been proposed to overcome disadvantages such as low frame rate and the flexible structure of the GI tract. These methods assist in keeping track of distinct points in successive frames, thereby aiding in estimating the capsule’s movement and orientation. The study [82] explores using PWC-Net, a deep learning model, to estimate optical flow, which shows the point motion between frames. A feature point tracking-based localization technique has been presented in [83], in which the Speeded-Up Robust Features (SURF) algorithm, combined with a Random Sample Consensus (RANSAC) approach, is used to detect and match these feature points, filtering out erroneous matches. The study evaluated the performance of a model by comparing the reconstructed paths of a capsule through the large intestine, resulting in an average path difference of 4 ± 0.7 cm. In addition, the study also proposed a frame classification system that helped to distinguish different components of the large intestine, and a mean accuracy of 86% was reported. Variable frame rates are utilized, of 4–35 Hz, that can help in the conservation of the battery in longer monitoring. Integrating the technique with deep learning models can be explored to enhance accuracy.

### 4.3. Summary of Video-Based Localization Techniques

New technologies demonstrated promising capabilities in localizing WCE by using video-based techniques. These techniques are further applied during the inspection to identify certain abnormalities, such as polyps. Several methods have been proposed regarding MEMS, such as multiplexing various components inside the capsule. Other techniques of dimensionality shifting are considered in order to enable more extensive image analysis after the WCE examination. In the last couple of years, the usage of machine learning algorithms for the improvement of features captured frames and proper localization of WCE has substantially increased from video data.

Real-time localization encounters problems with the capsule’s continuous movement inside the intestine. Real-time information on the orientation and motion of the capsule can be obtained through IMU data; this would be able to distinguish between peristaltic activity and that of the capsule’s motion. The method of video-based localization can be improved by updating the frame rate for efficiency in real applications. The increased frame rates can also be enabled by combining optimization algorithms with ML techniques to decrease the processing time. The integration of video frame localization with other localization methods and MEMS devices does offer a bright future for developing accurate real-time localization systems. Table 3 summarizes and compares some of the latest features in video-based localization for WCE.

## 5. Hybrid and Other Localization Methods

### 5.1. Introduction to Hybrid Localization Techniques

Recent advancements in miniaturized circuits, computational power, machine learning, and artificial intelligence have significantly improved localization results. High-quality, detailed, exhaustive datasets and modern techniques in data processing enable algorithms to produce localization solutions that are much more accurate and efficient. A strategic combination of different localization techniques, including magnetic, RF, and video-based localization, is necessary to address further limitations of these technologies. Hybrid methods typically integrate data from IMU sensors or RSSI values with video frames from the capsule to estimate its distance and orientation.

### 5.2. Emerging Trends in Hybrid Localization Techniques

In [84], a hybrid method is introduced in which the capsule’s orientation and direction of travel within the GI tract are determined using four low-resolution side-wall cameras and an IMU with a 9 DoF sensor that consists of a gyroscope, accelerometer, and magnetometer. Cameras capture capsule motion relative to GI tract walls, distinguishing capsule movement from intestine involuntary movements. The IMU unit measures the orientation, velocity, and gravitational forces acting on the capsule. An onboard microcontroller is used as the processing unit. The information is gathered from the IMU and the cameras, compiled into frames, and transmitted to an external data logger using a wireless connection. The novel fusion algorithm combines motion data from cameras and IMU to compute a precise trajectory of a capsule in three-dimensional space. It uses gyroscope data for short-term accuracy and accelerometer and magnetometer data for long-term stability by correcting drifting. The algorithm was evaluated experimentally by carrying out in-vitro validation on the porcine intestine, and an accuracy of 0.95 cm was reported. However, the prototype device has a large dimension, 3.5 cm × 3.5 cm × 4 cm, that can be reduced using current miniaturization techniques. The system requires a 94 mAh battery for 8 h of operation and requires optimization in order to achieve longer monitoring.

Similarly, in [86], a hybrid method combining magnetic and video imaging techniques is presented. The capsule is equipped with a small permanent magnet whose magnetic field is measured by nine three-axis Hall effect sensors positioned externally. The magnetic data are processed using mathematical models to reconstruct the capsule’s position, while low-resolution monochromatic side-wall cameras assess the capsule’s motion about the GI tract. MagnetOFuse is an adaptive algorithm that combines information available from magnetic and video sources, updating the weighting of the two in accordance with the motion to improve localization resolution. Experiments with the 3 × 3 sensor belt, an external robotic arm, and a workstation reported average positioning errors of around 0.84 mm when the capsules were stationary and 3.5 mm when they were in motion.

**Figure 6:**
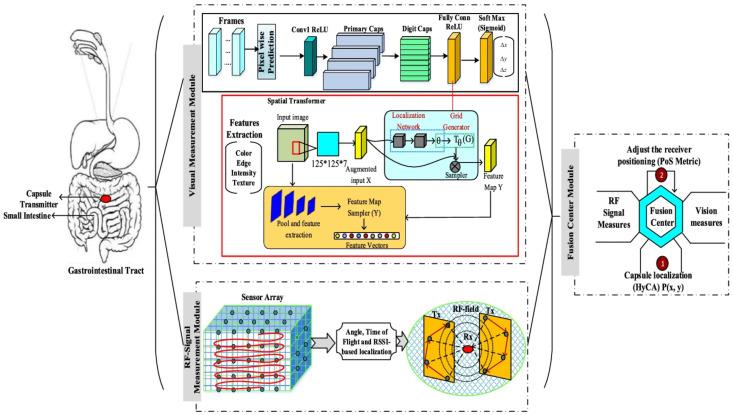
Overview of an RF and video-based hybrid localization technique [85].

The use of sophisticated machine learning methods and optimization algorithms allows the creation of dependable and accurate localization systems surpassing single-method-based simple scenarios. In [85], a new hybrid RF with a Vision-aware Fusion scheme, RF-VaF, is proposed. RF-VaF is a hybrid scheme combining the RF-based approach with video-based localization techniques. The localization is performed by using ToF and RSSI. The vision-based approach through a Siamese Capsule Network (CapsNet) and Spatial Transformer Network (STN) provides frame registration, mapping, and prediction (see Figure 6). A Hydrological Cycle Optimization (HCO) algorithm is proposed to enhance accuracy and reduce localization errors. Simulations with the RF-VaF scheme through human body modeling using the UWB channel path loss model and consecutive frames of WCE were conducted to evaluate this scheme. The mean localization error is reported as 5.41 mm, along with an overall accuracy of 96.43%.

In [87], an approach to improve the data transmission and localization accuracy of a four-camera VGA-resolution WCE system is presented to provide 360° image acquisition. A low-power, high-speed dual-band pulse-shaping transmitter is developed for up to 80 Mb/s data transmission. The L-M algorithm is implemented for the capsule tracking, and the contact attenuation compensated-received signal strength indicator (CAC-RSSI) algorithm is used to improve the accuracy. The system was experimentally evaluated using the pig intestine and human phantom, and an average localization error of 0.98 cm was reported. The capsule weighs less than 4 g and has a dimension of 32 mm × 12 mm. A 4 fps operation can be supported for 8 h with 55 mA.h of two coin batteries. Developing energy-efficient components and incorporating adaptive compression algorithms that dynamically balance image quality and power consumption based on real-time requirements can be explored to enhance the functionality of such systems.

Other techniques like medical and radiological imaging methods exist that do not have the possibility of being used with WCE or at least not as desired. Magnetic Resonance Imaging (MRI) or X-ray-based methods offer some high localization accuracy, but they remain limited by the radiation exposure itself. Computer Tomography has an accuracy in the sub-mm range, but it is highly invasive and not viable for any use in real-time tracking through surgery or examinations. Similarly, ultrasound-related methods pose risks of radiation exposure, particularly if one considers the mean duration of examination for the GI tract as taking between 8 and 12 h.

### 5.3. Summary of Hybrid Localization Techniques

With advancements in MEMS, nanotechnology, and computationally powerful devices, promising new developments are emerging for hybrid WCE localization methods. Recent studies have shown that error localization can now be obtained within the sub-mm range for practical real-time systems. The integration of different technologies enables multi-beneficial feature exploitation. It helps to overcome the limitations inherent in individual approaches, permitting surgeons to view and inspect the GI tract in more detail. For instance, IMUs can support real-time orientation data for the capsule and can be synchronized with video frames for finding and tracking any anomalies in the trajectory of the capsule. Similarly, the integration of various magnetic methods with RF-based techniques, augmented by various optimization algorithms, will increase localization accuracy.

However, the addition of more hardware within the WCE device increases the capsule beyond the standard dimension. Although multiple research works show that ASICs will be useful in minimizing the overall capsule size, high battery consumption still remains a challenge when the supplementary cameras, sensors, and units for processing are turned on [84,87]. Hybrid localization techniques that require additional hardware and sometimes onboard computing can be explored with WPT methods. Real-time location updates and precise localization information for a practical system are crucial. It would be interesting to use AI- and ML-based models with different optimization algorithms to reduce the update time further. The same can be used to minimize dependency on additional IMU and cameras inside the capsule to save volume for surgical purposes. Table 3 includes some of the latest advancements in the field of hybrid localization methods for WCE.

## 6. Conclusions

Endoscopy is a medical procedure that uses an endoscope to visually inspect the body’s internal organs. The demand for endoscopy procedures is on an upward trajectory due to the rise in the geriatric population and chronic diseases. WCE is a painless and non-invasive method of examining internal organs using a small camera swallowed like a pill. However, existing active locomotion technologies lack a practical localization system to securely manage the capsule’s movement within the body. We have identified salient features of different methods and categorized studies in tables. The review includes brief introductions of the latest methodologies used in the recent articles, the study environment, the type of hardware used, the reported localization error, and the way forward. In particular, the contributions of this work include a comprehensive updated review of magnetic, video, RF, and hybrid technology-based localization techniques.

The emerging technologies have shown promising results in accurately localizing WCE using static or dynamic magnetic fields, achieving position and orientation errors under 5 mm and 5°, respectively. Techniques often involve induction coils, IMU, and tri-axial sensors within the capsule. However, the limited volume of commercial capsules poses a significant constraint. WPT can be a promising solution for power transfer, estimating the capsule’s location, overcoming the limitations of traditional internal batteries, and enabling thorough, extended diagnostic procedures. Furthermore, the use of additional sensors, either within the WCE or in the localization system, can improve localization accuracy but may make the system impractical.

The RF-based localization system leverages the properties of the wireless propagation channel, including attenuation, relative permittivity, time, and multipath characteristics. Recently, there has been a growing emphasis on exploring various combinations of optimization algorithms, AI, circularly polarized antennas, arrays of antennas on the receiving side, and ML techniques to minimize localization errors. However, technical challenges, such as the need for line-of-sight, complicate the implementation of these technologies. Additionally, the body’s movement and the involuntary motion of internal organs contribute to higher localization errors. Implementing ToF, ToA, DoA, and AoA methods in real-time settings is much more challenging due to the high speed of EM waves. Furthermore, RF-based methods cannot determine the orientation of the capsule. RF-based techniques can localize the capsule without any additional hardware, and along with video or magnetic field-based techniques, the capsule’s orientation could also be determined.

Several methods have been proposed for video-based techniques, such as dimensionality shifting, to enable more extensive image analysis after the WCE examination. In the last couple of years, the usage of machine learning algorithms to improve the features captured in frames and proper localization of WCE has substantially increased from video data. However, real-time localization encounters problems with the capsule’s continuous movement inside the intestine. Real-time information on the orientation and motion of the capsule can be obtained through IMU data; this would be able to distinguish between peristaltic activity and that of the capsule’s motion. The method of video-based localization can be improved by updating the frame rate for efficiency in real applications. The increased frame rates can also be enabled by combining optimization algorithms with ML techniques to decrease the processing time. The integration of video frame localization with other localization methods and MEMS devices does offer a bright future for developing accurate real-time localization systems.

With advancements in MEMS, nanotechnology, and computationally powerful devices, promising new developments are emerging for hybrid WCE localization methods. The integration of different technologies enables multi-beneficial feature exploitation. It helps to overcome the limitations inherent in individual approaches, permitting surgeons to view and inspect the GI tract in more detail. It would be interesting to use AI and ML-based models with different optimization algorithms to reduce the update time further. The same can be used to minimize dependency on additional IMU and cameras inside the capsule to save volume for surgical purposes.

Future research should focus on using a minimal number of sensors and localization equipment inside the capsule and in the surrounding environment while emphasizing efficient and real-time algorithms. Recent advancements in onboard sensing demonstrated that compact size, low power consumption, and higher monitoring time are achievable. Moreover, wearable localization systems are highly desirable as they can assist patients in daily activities during extended examinations lasting up to 12–14 h. A robust, real-time, and practical localization system is essential to advance WCE and make it desirable for clinical trials. For real-time localization, minimizing computation time is crucial. Advanced machine learning techniques and various algorithm combinations can help to reduce computational time for effective real-time monitoring. This review presents an in-depth review of the most recent localization techniques published in the past 5 years, including magnetic field, RF, video, and hybrid-based techniques. This review can help researchers to understand the latest methods and identify potential areas for further investigation. 

## Figures and Tables

**Table 1:** Emerging magnetic-based localization strategies.

Ref (Year)	Technique/ Algorithm	WC, EC	Validation Environment	Error/Accuracy	Notes
[27] (2023)	Active Locomotion/ Jacobian-based iterative method	WC: IMU + 3-axis magnetic field sensor, EC: External PM	Experimental evaluation: Different translational and rotational motion	Average Positional Error (PE): 6.29 mm, Average Orientation Error (OE): 2.93°	6 DoF reported and magnetic moment is optimized for the magnetic dipole model.
[28] (2022)	Active Locomotion/direct estimation + Kalman filter	WC: IPM + BLE + IMU EC: 5 × 5 Hall sensors	Experimental evaluation: work ranges from 25 to 72 mm	Mean AE: 1.46 mm, Mean OE: 0.41°	6 DoF reported and, instead of using a PM, a magnetic capsule shell is proposed.
[29] (2020)	Passive Locomotion/Jacobian matrix	WC: IPM, EC: 4 triple-axis sensors	Experimental evaluation	Mean PE: 2.1 ± 0.8 mm, Mean OE: 6.7 ± 4.3°	Triple-axis sensors were utilized with the Jacobian method to achieve 5 DoF localization.
[31] (2023)	Active Locomotion/PSO + L-M	WC: IPM EC: Hall effect sensors	Experimental evaluation: static and dynamic	Mean AE: 1.46 mm, Mean OE: 0.41°	Fast tracking and 6 DoF localization are reported by combining the algorithms.
[32] (2019)	Passive Locomotion/ Variance-based algorithm	WC: IPM EC: 16 Hall effect sensor + IMU	Experimental evaluation: static capsule	Average PE: 9.73 mm, Average OE: 12°	The variance-based algorithm combined with weighted optimization is used to achieve 6 DoF localization.
[33] (2022)	Passive Locomotion/Fusion algorithm	WC: IPM EC: 36 Hall effect sensor + IMU	Experimental evaluation	Average PE: 1.8 mm, Average OE: 5.11°	The fusion algorithm calculates the quaternion rotation and 6 DoF localization is reported.
[34] (2021)	Active Locomotion/L-M + differential signals	WC: IPM EC: 16 tri-axis sensors	Experimental evaluation: static and dynamic motion	PE: 7.5 mm, Average OE: 13.8°	A symmetrically arranged cell of four sensors combined with different algorithms are used to achieve 6 DoF localization.
[35] (2022)	Passive Locomotion/L-M	WC: IPM EC: 12 Hall effect sensors	Experimental evaluation with different magnets	Relative PE: 4.3 ± 3.3 mm, Relative OE: 2 ± 0.6°	Neodymium N52 cylindrical permanent magnets with different diameters are used to achieve 6 DoF localization.
[36] (2021)	Active Locomotion/Differential method	WC: IPM EC: 8 Hall effect sensors	Experimental evaluation: multi-point simultaneous tracking	Average PE: 4.06 ± 0.29 mm, Relative OE: 5.63 ± 4.24°	The reported calculation time is 80 ms, and the algorithm can compensate for patients’ movements for 5 DoF localization.
[40] (2022)	Passive Locomotion	WC: IPM EC: 12 sensors, + orthogonal coils	Experimental evaluation: Dynamic magnetic field	Mean PE: 3.8 ± 1.1 mm, Maximum OE: 3°	The system used two orthogonal reference coils alternately switching on and off with a low switching speed for 6 DoF localization.
[41] (2021)	Active Locomotion/RMSD	WC: IPM + tri-axial sensors EC: 4 electromagnets	Experimental evaluation: Dynamic and static magnetic fields	Position accuracy (PA): 5 mm, Orientation accuracy (OA) 5°	The optimization process utilized a dual-step approach for 6 DoF localization.
[45] (2021)	Active Locomotion/Linear prediction	WC: RX coils EC: TX coils	Experimental evaluation: Dynamic magnetic field	PE: 12 mm	A dual-purpose use of WPT, not only for powering the capsule, but also for 3 DoF localization within the GI tract, is proposed.
[46] (2024)	Passive Locomotion/LSE, SD	WC: Orthogonal Coils EC: TPT	Experimental: VNA Measurement	Accuracy < 1 cm	The study utilizes QS-MI for precise localization, and the method is validated through simulation and VNA measurements.
[47] (2020)	Active Locomotion/L-M + PSO	WC: Induction coil EC: Electromagnets	Experimental evaluation: Dynamic and static magnetic fields	Average PE: 2.3 mm, Average OE 0.2°	An innovative feature of the methodology is the use of nine-channel sinusoidal signals to stimulate the transmitting coils for 6 DoF localization.
[48] (2024)	Passive Locomotion/L-M	WC: Induction coil + AFE + Modulator EC: 8 TX coils	Experimental evaluation: Dynamic magnetic fields	PA: 0.8 mm, OE 1.1°	On-chip sensing method utilizing CMOS 65 nm technology for a compact and cheap design for 5 DoF localization.
[51] (2023)	Passive Locomotion/FDMML + Welch’s method + ANNs	WC: Induction coil + wireless TX + battery EC: 6 TX coils	Experimental evaluation: Dynamic magnetic fields	PA: <1mm	The FDMML technique assigns unique offset frequencies to external magnetic beacons, allowing them to operate simultaneously and eliminating the need for sequential activation.

**Table 2:** Recent developments in RF-based localization.

Ref (Year)	Technique	Algorithm	Validation Environment	Error/Accuracy	Notes
[56] (2023)	UWB RSSI	LWLR and k-fold cross validation	Simulation: 8 and 48 RXs	RMSE less than 0.23 mm	The results from different algorithms were optimized at UWB and MICS bands.
[57] (2022)	RSSI	Trilateration	High-Definition Numerical Human Body Model	Simulation Accuracy: 92% Experimental Accuracy: 89.7%.	The method incorporates RSSI magnitude pattern and antenna angle error correction techniques at 433.92 MHz.
[58] (2021)	RSSI	SVM and Moore–Penrose	Simulation: 10 RXs	Error: 2 mm	The study uses an electromagnetic scattering model at 17 MHz for localization.
[60] (2024)	RSSI	Trilateration, SNN	Simulation: Human model	Error: 26.44 mm	The study combines one-shot learning and trilateration methods at 4 GHz.
[61] (2022)	RSSI	WCL	Experimental: 24 antenna array	Error: 21.9 mm	The WCL algorithm applies exponential weights to RSSI values at 433 MHz.
[63] (2022)	PDoA	Gauss–Newton, Phase detection algorithm	Simulation: Remcom XFdtd Software, Experimentation: helical antenna	Average Error: 16 mm	The accuracy is reportedly improved by approximately 30% when using a combination of the helical antenna and phase detection algorithm compared to a homogeneous body model at MICS band.
[65] (2020)	PDoA	Gauss–Newton, Phase detection algorithm	Simulation: Remcom XFdtd Software, Experimentation: half-wave dipole	MSE: 28 mm	The accuracy is reportedly improved by approximately 15% when using an adaptive simplified human body model compared to a homogeneous body model at MICS band.

**Table 3:** Video-based and hybrid localization techniques.

Ref (Year)	Technique	Algorithm Environment	Validation	Error/Accuracy	Notes
[77] (2022)	Image Processing with a self-attention mechanism	Attention Aware CNN	Public datasets: Bleeding dataset and Kvasir-Capsule dataset	Accuracy: Bleeding dataset: 95.1% Kvasir-Capsule dataset: 94.7%.	A dual-branch CNN model integrating self-attention mechanisms and using ResNet-50 to improve classification accuracy and lesion localization in WCE images at 30 fps.
[79] (2019)	Modified R-CNN	ResNet-50 and ResNet-101 models with data augmentation and fine-tuning	CVC-ColonDB, CVC-PolypHD, and ETIS-Larib	F1 score: 96.67% F2 score: 96.10%.	The work introduces a modified R-CNN for polyp identification and adapts deep learning models trained on non-medical images.
[80] (2021)	Deep CNN with attention mechanism	WCENet Grad-CAM++ and SegNet	KID dataset for WCE images	Accuracy: 98%, Dice Score: 56%	The study introduces a hybrid anomaly localization method for identification and segmentation of abnormal regions.
[83] (2021)	Feature point tracking techniques	SURF and RANSAC	84 videos from 42 patients	Error: 4 ± 0.7 cm	The study utilizes feature point tracking to estimate capsule displacement and orientation.
[84] (2021)	Hybrid: Video + IMU	Fusion Algorithm	Experiment: Ex-vitro porcine intestine	Accuracy: 0.95 cm	Hybrid method uses four low-resolution side-wall cameras and an IMU with a 9 DoF sensor for 6 DoF localization.
[85] (2022)	Hybrid: Video + RSSS + ToF	STN, HCO, CapsNet	Simulation: UWB, 8–50 RXs	Error: 5.41 mm Accuracy: 96.43%	The method integrates RF and vision-based data for localization using a fusion of multiple algorithms.
[86] (2022)	Hybrid: Video + Magnetic	MagnetO Fuse	Experiment: 3 × 3 sensor array, robotics arm, bio-tissues	Average Error: Stationary Capsule: 0.84 mm Moving Capsule: 3.5 mm	The proposed algorithm uses mathematical models to reconstruct the capsule’s position and low-resolution side wall cameras to assess motion.
[87] (2018)	Hybrid: Video + RSSI	CAC-RSSI, L-M	Experiment: Human mimicking phantom and pig small intestine	Error: 0.98 cm	A four-camera VGA-resolution WCE system is used to improve data transmission and localization accuracy, utilizing BCC, CAC-RSSI, and L-M algorithms.
